# Pneumonic Plague Outbreak, Northern Madagascar, 2011

**DOI:** 10.3201/eid2101.131828

**Published:** 2015-01

**Authors:** Vincent Richard, Julia M. Riehm, Perlinot Herindrainy, Rahelinirina Soanandrasana, Maherisoa Ratsitoharina, Fanjasoa Rakotomanana, Samuel Andrianalimanana, Holger C. Scholz, Minoarisoa Rajerison

**Affiliations:** Institut Pasteur de Dakar, Dakar, Senegal (V. Richard);; Bundeswehr Institute of Microbiology, Munich, Germany (J.M. Riehm, H.C. Scholz);; German Center for Infection Research, Munich (J.M. Riehm, H.C. Scholz);; Institut Pasteur de Madagascar, Anatananarivo, Madagascar (P. Herindrainy, R. Soanandrasana, M. Ratsitoharina, F. Rakotomanana, M. Rajerison);; Ministry of Public Health, Anatananarivo (S. Andrianalimanana)

**Keywords:** pneumonic plague, outbreak, Yersinia pestis, bacteria, drug resistance, pathogenicity, typing, epidemiology, zoonoses, Madagascar

## Abstract

*Yersinia pestis*, the causative agent of plague, is endemic to Madagascar, particularly to the central highlands. Although plague has not been previously reported in northern Madagascar, an outbreak of pneumonic plague occurred in this remote area in 2011. Over a 27-day period, 17 suspected, 2 presumptive, and 3 confirmed human cases were identified, and all 15 untreated 20 patients died. Molecular typing of *Y. pestis* isolated from 2 survivors and 5 *Rattus rattus* rat samples identified the Madagascar-specific 1.ORI3-k single-nucleotide polymorphism genotype and 4 clustered regularly interspaced short palindromic repeat patterns. This outbreak had a case-fatality rate of 100% for nontreated patients. The *Y. pestis* 1.ORI3-k single-nucleotide polymorphism genotype might cause larger epidemics. Multidrug-resistant strains and persistence of the pathogen in natural foci near human settlements pose severe risks to populations in plague-endemic regions and require outbreak response strategies.

## Abstract 2

Multidrug-resistant *Yersinia pestis* is a serious threat that requires outbreak response strategies.

*Yersinia pestis* is the causative agent of plague, a severe and life-threatening zoonotic disease. During 3 pandemics, different genotypes of this bacterium spread to various countries and caused millions of deaths (*1*–*6*). *Y. pestis* genotype 1.ORI3 of the biovar Orientalis was introduced to Madagascar in 1898 during the third pandemic and has persisted there endemically ever since (*6**,**7*). Worldwide, an average of 4,000 human plague cases is reported each year. Madagascar is one of the most active plague foci and has an annual average of 1,500 confirmed cases (*8*,*9*). The pathogen emerges in multiannual cycles; a peak of pathogen prevalence was observed in Madagascar in 1997 and caused ≈3000 cases, 20 times more than in 1994 (126 cases) (*9*).

Bubonic plague, the most common form of plague, results from the bite of an infected flea. The infection may spread hematogenously and cause secondary pneumonic plague. If the pathogen is transmitted as an aerosol by droplets or by contaminated dust, primary pneumonic plague may result. After a latency period of 1–5 days, pneumonic plague progresses to the stage of hemoptysis. At this lethal stage of the disease, which lasts ≤3 days, patients are highly infectious (*10*,*11*). Plague can be treated with antimicrobial drugs if diagnosed early and if caused by a drug-sensitive strain (*9*).

Worldwide, only a few pneumonic plague outbreaks have been reported (*12*–*15*). However, to understand the epidemic potential of *Y. pestis,* extensive outbreak analyses are essential. In 1997, a pneumonic plague outbreak occurred in Madagascar near the capital of Antananarivo (*13*). Health authorities responded immediately, and strain cultivation was successful. Three other pneumonic plague outbreaks have been reported, 1 in Uganda (2004) and 2 in the Democratic Republic of Congo with 87 cases (2005) and 117 cases (2006), respectively (*14*,*15*). During the 1920s–1930s, valuable descriptions of 2 plague epidemics in Manchuria, China (≈10,000 cases) were reported (*10*,*12*).

In this report, we describe an outbreak and highly progressive spread of pneumonic plague in northern Madagascar, a remote region that was supposedly free of *Y. pestis*, in 2011. We investigated whether *Y. pestis* might cause larger outbreaks or epidemics with high case-fatality rate within a short period.

## Outbreak Progression and Investigation

**Figure 1:**
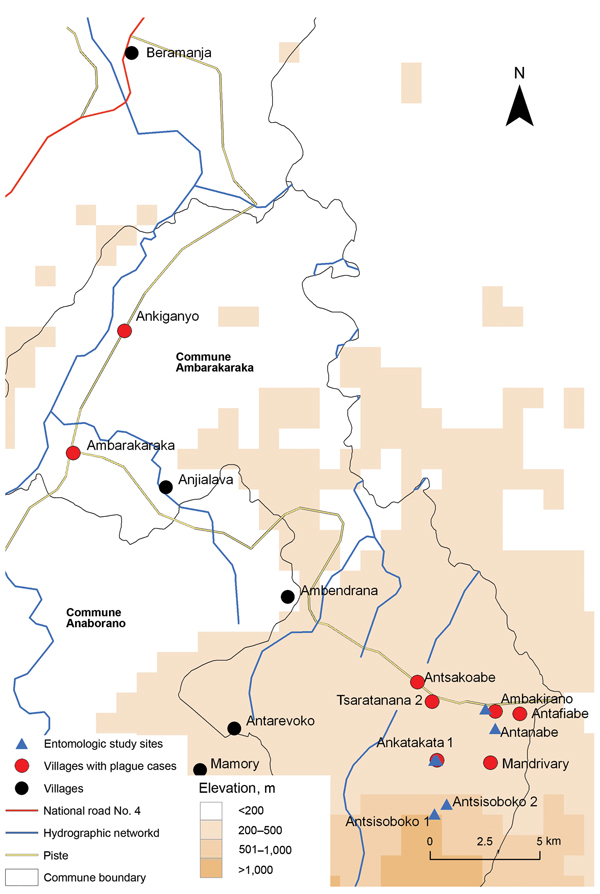
Location of pneumonic plague outbreak in the communes of Ambarakaraka and Anaborano, northern Madagascar, 2011. A copper mine is located in Beramanja. The index case-patient was infected with *Yersinia pestis* on the 80-km trail (piste) to Ankatakata.

**Figure 2:**
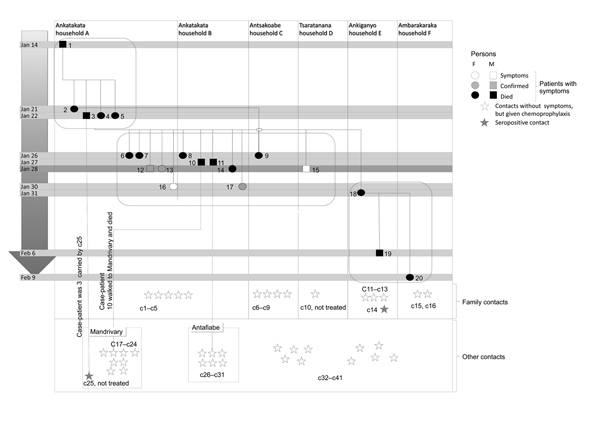
Infection pattern during pneumonic plague outbreak, northern Madagascar, 2011. The outbreak spread to other neighboring villages during January 14–February 9. Twenty persons in 6 households (A–F) in 5 villages had symptoms of pneumonic plague. The outbreak population was divided into 3 groups (group 1: case-patients 1–5; group 2: case-patients 6–17; and group 3: case-patients 18–20). Patients received treatment by January 28. Because of geographic distance, none of the patients in group 3 received treatment. Contacts were divided into family contacts (c1–c16) who lived in an affected household and other contacts (c17–c41) who interacted with infected patients or patients who died. All contacts, except c10 and c25, received antimicrobial drug prophylaxis. Two contacts (c14 and c25) were seropositive (single serum sample); all other contacts remained seronegative.

The outbreak investigation protocol was approved by the Ethical Committee, Ministry of Health of Madagascar. The outbreak area contained 7 villages along a field path in the communes of Ambarakaraka and Anaborano, Ambilobe District, in northern Madagascar at an altitude <500 m. In January 2011, two brothers (boys) were working in a copper mine in Beramanja, Madagascar. On January 6, the boys returned home to Ankatakata (distance 50 km) (Figure 1). During the journey, fever, headache, and chills developed in 1 of the boys (age 13 years). Subsequently, he experienced severe chest pain, a deep cough, and hemoptysis, which are the clinical signs of pneumonic plague. He died at home on January 14 (Figure 2). It can be assumed retrospectively that this boy (index case-patient 1) had been infected with *Y. pestis* in Beramanja or during the journey home (Figures 1,2). On January 21, his mother, who had provided him with treatment until he died, also died (case-patient 2 in household A). Her husband (case-patient 3), daughter (case-patient 4), and granddaughter (case-patient 5) died on January 22. Symptoms of pneumonic plague had developed in these 3 persons (Figure 2; Table 1). These 3 persons were provided treatment by neighbors in household B (Figure 2).

**Table 1:** Diagnostic, epidemiologic, and molecular data for persons with symptoms of pneumonic plague during pneumonic plague outbreak, northern Madagascar, 2011*

Patient/household	Received antimicrobial drug treatment and survived	Sampling	RDT	Serologic analysis	Culture	WHO case definition	Molecular analysis
1/A	No	No	ND	ND	ND	Suspected	ND
2/A	No	No	ND	ND	ND	Suspected	ND
3/A	No	No	ND	ND	ND	Suspected	ND
4/A	No	No	ND	ND	ND	Suspected	ND
5/A	No	No	ND	ND	ND	Suspected	ND
6/A	No	No	ND	ND	ND	Suspected	ND
7/A	No	No	ND	ND	ND	Suspected	ND
8/B	No	No	ND	ND	ND	Suspected	ND
9/C	No	No	ND	ND	ND	Suspected	ND
10/B	No	No	ND	ND	ND	Suspected	ND
11/B	No	No	ND	ND	ND	Suspected	ND
12/A	Yes	Serum/sputum	+	+†	–	Confirmed	+
13/A	Yes	Serum/sputum	+	+†	–	Confirmed	+
14/B	No	No	ND	ND	ND	Suspected	ND
15/D	Yes	Serum	ND	–	ND	Suspected	ND
16/A	Yes	Serum	ND	–	ND	Suspected	ND
17/B	Yes	Serum	ND	+†	ND	Confirmed	ND
18/E	No	No	ND	ND	ND	Suspected	ND
19/E	No	No	ND	ND	ND	Suspected	ND
20/F	No	No	ND	ND	ND	Suspected	ND

*RDT, rapid dipstick test; WHO, World Health Organization; ND, not done; +, positive; –, negative. †A 4-fold increase in titer in the second serum sample.

On January 26, a second wave of human pneumonic plague cases was observed in 12 other persons (Figure 2; Table 1). During this second wave, household A (case-patients 6, 7, 12, 13, and 16) and household B (case-patients 8, 10, 11, 14, and 17) in Ankatakata were affected. Case-patient 9 was probably infected while visiting her sister-in-law (case-patient 2) in Ankatakata and transmitted the pathogen to her home (household C) in Antsakoabe (Figures 1,2). Case-patients 15 and 18 were also present in Ankatakata and transmitted the infection to Tsaratanana (household D) and Ankiganyo (household E). Case-patient 18 was responsible for a third small wave of pneumonic plague that started on January 31 and spread to Ambarakaraka (Figure 2; Table 2). This case-patient infected her brother (case-patient 19), who had carried his sister to a traditional healer (case-patient 20). Symptoms of pneumonic plague developed in all 3 persons; all 3 died (Figure 2; Table 1).

**Table 2:** Characteristics of 41 contacts and plague patients during pneumonic plague outbreak, northern Madagascar, 2011*

Contact/relationship	Symptoms	Chemoprophylaxis	Serologic result†	WHO case definition	Village	Characteristic
1–5, family	None	Yes	–	NA	Ankatakata	Houses A and B
6, family	None	Yes	–	NA	Antsakoabe	House C, husband of patient 9
7, family	None	Yes	–	NA	Antsakoabe	House C, children of patient 9
8 and 9, family	None	Yes	–	NA	Antsakoabe	House C
10, family	None	No	–	NA	Tsaratanana	House D
11–13, family	None	Yes	–	NA	Ankiganyo	House E
14, family	None	Yes	+	Presumptive	Ankiganyo	House E, mother of patient 18
15 and 16, family	None	Yes	–	NA	Ambarakaraka	House F
17–24, other	None	Yes	–	NA	Mandrivary	Met with patient 10
25, other	Cough	No	+	Presumptive	Mandrivary	Carried patient 3
26, other	None	Yes	–	NA	Antafiabe	Shared bed with patient 11
27–31, other	None	Yes	–	NA	Antafiabe	Met with patient 11
32–34, other	Mild	Yes	–	NA	Several	Attended funerals
35–41, other	None	Yes	–	NA	Several	Attended funerals

*Contacts of plague patients were divided into family contacts (persons who lived in the same household with an infected person) and other contacts (persons spent time with a patient or approached a persons who died). WHO, World Health Organization. †For *Yersinia pestis*.

### Contacts

During our epidemiologic investigation, 41 contact persons were identified. These persons had interacted with the patients but did not show specific symptoms of pneumonic plague. All provided a serum sample for diagnostic testing (Table 2). Sixteen family contacts (c1–c16) were defined as persons who lived in the same household as an infected person during the outbreak (Figure 2; Table 2). Twenty-five other contacts (c17–c41) had spent some time with a patient or approached a patient who died during the outbreak (Figure 2; Table 2).

In Ankatakata, we found 5 family contacts (c1–c5) in households A and B who had received chemoprophylaxis on January 28. In Antsakoabe, case-patient 9 died in her home on January 26. Her husband and 3 children (c6–c9) lived in the same house. They received chemoprophylaxis on February 6 and provided serum samples on February 15. In Tsaratanana, the wife (c10) of case-patient 15 refused chemoprophylaxis. In Ankiganyo, 4 family contacts (c11–c14) of deceased case-patients 18 and 19 received chemoprophylaxis on February 6. Contact c14 was seropositive for pneumonic plague. In Ambarakaraka, 2 family contacts (c15 and c16) of case-patient 20 received chemoprophylaxis on February 9 (Figure 2; Table 2).

The status of nonfamily contacts was as follows. Case-patient 10 moved to Mandrivary, which is 8 km from Ankatakata, and died there of suspected pneumonic plague. We identified 9 contact persons (c17–c25) in Mandrivary. All except 1 (c25) had received chemoprophylaxis on January 28 and showed seronegative results. Contact 25 was a 21 year-old man who had carried case-patient 3 in Ankatakata. He had a cough and was seropositive (Table 2; Figure 2). Case-patient 11 died in Antafiabe. He had contact with 6 other persons (c26–c31). These 6 persons received chemoprophylaxis on January 28. Although 1 contact (c26) shared her bed with case-patient 11 until his death on January 27, these 2 persons did not show clinical signs or symptoms or seroconversion. We found 10 additional contacts (c32–c41), who had attended funerals for case-patients in different villages. Three of these contacts had mild pulmonary infection, but had not consulted a physician and were seronegative (Figure 2; Table 2).

### Diagnosis and Case Assessment

Two weeks after the death of the index case-patient on January 28, health personnel arrived in Ankatakata. Treatment with antimicrobial drugs was provided to 5 patients (case-patients 12, 13, 15, 16, and 17), and chemoprophylaxis was provided to contact persons and health personnel according to recommendations of the World Health Organization (WHO) (*9*) (Figure 2; Tables 1, 2). Because we did not know that the outbreak was ongoing in Ankiganyo (30 km from Ankatakata), treatment was not provided to case-patients 18, 19, and 20 (Figures 1, 2). It was only after these 3 case-patients died that contacts of these patients received chemoprophylaxis (Figure 2; Table 2).

Because persons who died were not sampled postmortem, only a limited number of clinical samples were collected (Table 1). Of collected sputum samples, 2 (from case-patients 12 and 13) were positive for *Y. pestis*–specific F1 antigen by immunochromatographic rapid dipstick test (Table 1) (*16*). All samples were subsequently transported (>900 km) to the WHO Collaborating Center for Plague at the Institut Pasteur de Madagascar in Antananarivo. Sputum samples were incubated in bacterial culture media and inoculated into laboratory mice. However, isolation of *Y. pestis* was not successful (Tables 1,3). Collected serum samples were analyzed by using a *Y. pestis*–specific F1 antigen IgG ELISA as described (*17*). Positive samples were quantified by using serial dilutions. Three of 5 paired serum samples (from case-patients 12, 13, and 17) had 4-fold increases in titer in the second serum (Table 1). Of samples from contact persons, 2 of 41 single serum samples showed a positive result (Table 2).

**Table 3:** Analysis of animal serum samples and organs for *Yersina pestis*, April 1, 2011, during pneumonic plague outbreak, northern Madagascar, 2011*

Village	Rodent or dog source	No. samples	Serologic result	*pla/caf1 *PCR result	Culture	SNPs	CRISPR	Sample ID
Antanabe	*Rattus rattus*	1	–	+	–	+	+	R05
	*R. rattus*	20	–	–	ND	ND	ND	NA
	*Mus musculus*	1	–	–	ND	ND	ND	NA
	*Suncus murinus*	1	–	–	ND	ND	ND	NA
Ambarakaraka	*R. rattus*	1	–	+	–	+	+	R16
Ankatakata	*R. rattus*	1	–	+	–	+	+	R48
	*R. rattus*	1	–	+	–	+	+	R52
	*R. rattus*	1	–	+	–	+	+	R56
	*R. rattus*	15	–	–	ND	ND	ND	NA
	*Canis* sp.	2	+	ND	ND	ND	ND	NA
	*Canis* sp.	3	–	ND	ND	ND	ND	NA
	*M. musculus*	2	–	–	ND	ND	ND	NA
	*Setifer setosus*	1	+	–	ND	ND	ND	NA
Antsisoboko	*R. rattus*	2	–	–	ND	ND	ND	NA
	*S. setosus*	2	–	–	ND	ND	ND	NA
	*Microgale brevicaudata*	3	–	–	ND	ND	ND	NA
Antanambao	*R. rattus*	9	–	–	ND	ND	ND	NA
	*S. setosus*	3	–	–	ND	ND	ND	NA
Total	NA	69	3 positive	5 positive	NA	5	5	NA

*pla, plasminogen activator gene; *caf1*, capsule antigen fraction1 gene; SNPs, single-nucleotide polymorphisms; CRISPR, clustered regularly interspaced short palindromic repeat pattern; ID, identification; –, negative; +, positive; ND, not done; NA, not applicable.

Subsequent case assignment was conducted according to WHO recommendations for plague-endemic countries (*18*). Three types of cases were identified: suspected cases (specific clinical symptoms), presumptive cases (positive serologic result for antibody against F1 antigen), confirmed cases (4-fold increase in titer of antibody against F1 antigen in paired serum samples or a positive culture result) (*18*). When we applied these recommendations to the outbreak, we identified 17 suspected cases (in case-patients 1–11, 14–16, and 18–20), 2 presumptive cases (in c14 and c25), and 3 confirmed cases (in case-patients 12, 13, and 17) (Tables 1,2).

All 20 patients with pneumonic plague had sudden onset of fever, cough, hemoptysis, and chest pain. The latency period was 4–6 days, and the infectious period was 48–72 hours (Figure 2). When given antimicrobial drugs, 5 patients (case-patients 12, 13, 15, 16, and 17) survived (Table 1). In contrast, the 15 case-patients who were not treated died of pneumonic plague. Of the 36 persons living in affected households A–F, 20 showed specific symptoms and 15 died. The overall attack rate was 55%, and the case-fatality rate was 75% (Figure 2).

### Outbreak Focus

Because of poor accessibility to the remote area of the outbreak and thunderstorms, a field investigation on the plague focus was not started until April 1, which was 2 months after the outbreak (Table 3). No dead rats were observed before, during, and after the outbreak, which is an unusual finding for a plague epidemic in Madagascar.

The sampling sites were chosen in the 2 villages (Ambakirano and Ankatakata) and surrounding woodlands of Antsisoboko and Antanabe (Figure 1). A total of 36 traps were set during 30 nights. Sixty-four rodents were trapped: 51 black rats (*Rattus rattus*), 3 house mice (*Mus musculus*), 6 greater hedgehogs (*Setifer setosus*), 3 short-tailed shrews (*Microgale brevicaudata*), and 1 Asian house shrew (*Suncus murinus*). Serum or spleen samples were obtained from these 64 rodents and from 5 dogs (Table 3). Pathogens were not isolated from these animals. Fleas were not detected in flea-specific traps.

Bacteriologic culture, serologic analysis, and molecular testing were conducted as described for human samples. Bacteriological culture results was negative, but 1 *S. setosus* hedgehog and 2 dogs were seropositive for IgG against F1 by ELISA (Table 3).

### Molecular Investigation

Molecular diagnostics of *Y. pestis* DNA was performed by using a PCR specific for the *Y. pestis* plasminogen activator and capsule antigen fraction 1 genes as reported (*19*,*20*). Samples from case-patients 12 and 13 showed positive results for *Y. pestis* DNA (Table 3). For comparison, samples from person with confirmed human plague were also analyzed: 1 each from Mandritsara (2010) and Bealanana (2011) (400 km from Ambilobe) and 1 from Ankazobe (2010) (800 km from Ambilobe) (Table 4). Of animal spleen samples collected, 5 samples from *R. rattus* rats were positive for *Y. pestis* DNA (Table 3).

**Table 4:** Molecular typing of *Yersinia pestis* in 10 samples from humans and rats during pneumonic plague outbreak, northern Madagascar, 2011*

Sample origin	Source†	Distance, km‡	Year	*Ypb* locus	*Ypc* locus§
Ankatakata, case 12	Human	0	2011	b1-b2-b3-b4-b5	c1-c2-c3
Ankatakata, case 13	Human	0	2011	b1-b2-b3-b4	c1-c2-c3
Ankatakata #R48	Rat	0	2011	b1-b2-b3-b4-b5	c1-c2-c3
Ankatakata #R52	Rat	0	2011	b1-b2-b3-b4-b5	c1-c2-c3
Ankatakata #R56	Rat	0	2011	b1-b2-b3-b4-b5	c1-c2-c3
Antanabe #R05	Rat	3	2011	b1-b2-b3-b4-b5	NA
Ambarakaraka #R16	Rat	3	2011	b1-b2-b3-b4-b5	c1-c2-c3′-c12
Bealanana #013	Human	400	2010	b1-b2-b3-b4-b5	c1-c2-c3′-c12
Ankazobe #275	Human	400	2010	b1-b2-b3-b4-b5	c1-c2-c3
Mandritsara #438	Human	800	2011	b1-b2-b3-b4-b5	c1-c2-c3

*The s232 locus for all 10 isolates had a derived status, and the 13 single-nucleotide polymorphisms (s1362, s1375, s190, s197, s1367, s1004, s1025, s1377, s1089, s1363, s206, s1373, and s152) for all 10 isolates had an ancestral status. †Rat, *Rattus rattus*. ‡Distance to outbreak village (Ankatakata). §c3, 5′-CTGAAATACAAATAAAATAAATCGTCGAACAT-3′; NA, no amplification; c3′, 5 ′-CTGAAATACAAATAAAATAAATCGTCGAACA-3′; c12, 5′-ATCGAGGCGGGCCGGAAGAATGTCACGGCGGTT-3′.

We analyzed 14 canonical single-nucleotide polymorphisms (SNPs) to determine the SNP genotype (phylogenetic position) of 10 *Y. pestis*–positive samples within the 1.ORI3 group (s232, s1362, s1375, s190, s197, s1367, s1004, s1025, s1377, s1089, s1363, s206, s1373, and s152) according to a hierarchical molecular typing approach (*1*). All 10 samples showed an identical 1.ORI3-k SNP pattern; we also found 1 derived SNP (s232) and 13 ancestral SNPs (Table 4).

In a second typing approach, *Y. pestis*–specific clustered regularly interspaced short palindromic repeats (CRISPRs) were identified (*21*–*23*). Sequences obtained were compared with sequences reported (*21*–*23*) and those in the CRISPR database (http://crispr.u-psud.fr/). All 10 *Y. pestis*–positive samples had an identical *Ypa* locus (a1-a2-a3-a4-a5-a6-a7-a8). For the *Ypb* locus, 1 spacer reduction (b5) was detected in a sample from case-patient 13. The other 9 samples showed an identical *Ypb* locus (b1-b2-b3-b4-b5). Seven samples had an identical *Ypc* locus (c1-c2-c3). However, within this locus, a new, and to our knowledge, Madagascar-specific spacer c12 was detected in *R. rattus* rodent sample R16 from Ambarakaraka and in a Bealanana-013 sample from 2010 (Table 4). The newly found spacer sequence originated from a phage: c12: 5′-ATCGAGGCGGGCCGGAAGAATGTCACGGCGGTT-3′ (by BLAST analysis; http://blast.ncbi.nlm.nih.gov/Blast.cgi). The presence of spacer c12 shows a correlation with a 1-nt reduction in the precedent spacer (c3>c3′) in both samples (Table 4). Despite performing several PCRs, we could not amplify the *Ypc* locus from *R. rattus* rodent sample R05 from Antanabe (Table 4).

## Discussion

Three plague pandemics and numerous plague epidemics have been caused by *Y. pestis* in the past 1,500 years (*2*,*4*,*7*,*12*,*24*). During the second medieval pandemic, which also included the Black Death period, 50% (≈50 million persons) of the human population in Europe reportedly died of plague (*2*,*4*). At the beginning of the 20th century, 10,000 persons died during 2 plague epidemics in Manchuria, China (*12*). Although plague is still endemic to other countries, the reported numbers of plague patients has decreased to an average of 4,000/year since 1954. Also, there has been a slight decrease regarding the highest reported case number from countries in Asia and Africa (*8*,*9*).

Since the introduction of plague to Madagascar in 1898, the Institute Pasteur in Antananarivo was assigned to control this disease. The pneumonic plague outbreak in 2011 shows that despite introduction of education programs, a plan to investigate plague outbreaks promptly, and a trained task force, plague outbreaks cannot be prevented. However, in contrast to control of historical plague, when treatment with antimicrobial drugs was not available and the disease could spread unhindered, the present outbreak was stopped quickly after 27 days because of successful treatment with antimicrobial drugs (Figure 2). The good response of the patients to these drugs suggests that the *Y. pestis* strain that caused this outbreak was susceptible to streptomycin. However, streptomycin-resistant *Y. pestis* strains were isolated in Madagascar in 1995 and also during the outbreak in 2011 (*25*; M. Rajerison, pers. comm.). Therefore, drug-resistant *Y. pestis* strains may pose a new challenge to health authorities.

During the outbreak in 2011, persons shared single-room houses, lived in extended families, and closely cared for each other. Patients were not isolated. Because of social conventions, some persons trusted a traditional healer rather than physicians. This finding led to the third wave of the outbreak, which included 3 case-patients with pneumonic plague who died (Figure 2; Table 1).

WHO lists plague as a disease for which patients should be quarantined and requires that pneumonic plague patients are isolated from healthy persons (*9*). However, during the latency period before hemoptysis, sputum contains hardly any infectious organisms (*11*,*26*). Simple countermeasures, such as protective facial masks, are efficient in preventing transmission by droplets. Also, turning one’s head away from or turning one’s back toward a healthy person has a major prophylactic effect (*26*). This finding might explain why c26, who shared the same bed with case-patient 11 until his death, was not infected (Tables 1, 2). Thus, knowledge of the pathogenesis of *Y. pestis* in humans is essential for persons who live in plague-endemic countries.

It has been suggested that patients with bubonic plague and patients who have died of plague are not directly infectious to other humans (*9*,*26*). This suggestion is consistent with findings in the present study because contacts (c32–c41) who only attended the funerals did not show symptoms or seroconversion (Table 2; Figure 2).

Plague is endemic to Madagascar, especially in the central highlands (*7*). At an altitude >800 m, large numbers of rodent species and insectivores live in the rain forest. Those animals represent the classical natural focus for *Y. pestis* (*7*). The low-elevation seaport villages of Mahajanga and Antananarivo are exceptions to this altitude factor. Because of trade and stockpiling of grain and other food products, homophilous species, such as the black rat (*R. rattus*) and the Norwegian rat (*R. norvegicus*), play a major role in the urban lifecycle of plague (*6*,*7*,*27*). The present outbreak occurred at an altitude <500 m in a region that does not have much commercial or economic activity. Because of this finding, there was a low prevalence of small mammals in this area, and only a low number of rodents and insectivores were trapped during the epidemiologic investigation (Table 3). This factor resulted in the plague outbreak not being immediately recognized.

Although the outbreak *Y. pestis* strain could not be isolated, information was obtained by molecular analyses of human and animal samples. All samples contained the Madagascar-specific 1.ORI3-k genotype of *Y. pestis*, as previously reported (*1*,*6*). Results of CRISPR typing identified >1 genotype, which indicated that the outbreak area was a natural plague focus before the outbreak in 2011 (Table 4). This result is supported by an unusual high prevalence (12%, 8/69) of *Y. pestis*–positive animal samples (Table 3) compared with prevalences in previous studies (*28*–*30*). We suggest that *Y. pestis* strains containing the major CRISPR profile, which was found in 6 of 10 samples, was responsible for the present outbreak (Table 4). This CRISPR genotype has also been found in samples from the central highlands. The isolate from case-patient 13 lost the b5 spacer (Table 4). This phenomenon has been reported for other CRISPR profiles, and a different genotype has been assessed (*21*–*23*). A third *Y. pestis* CRISPR genotype was found in the *R. rattus* rodent R16 sample and in the human Bealanana 013 sample from 2010; this genotype includes the new element c3′–c12 of phage origin (Table 4) (*21*). The loss of 1 nt at the end of the spacer, as observed in in c3>c3′, has been previously reported (*22*).

Despite performing several PCRs, we could not amplify the complete *Ypc* locus in the *R*. *rattus* rodent R05 sample from Antanabe (Table 4), a finding that has been previously reported (*21*). We suggest that 4 CRISPR genotypes of *Y. pestis* were present in the outbreak area, which indicates that Ambilobe was a natural plague focus even before the outbreak. Unnoticed presence of pathogens near human populations requires higher surveillance activity, as recently reported (*7*). In contrast, it has been reported that hereditary resistance against *Y. pestis* might develop in rats (*7*,*31*).

One question that also needs to be addressed is why plague caused millions of deaths during devastating pandemics in the past while today plague is restricted to some geographic locations. One possible explanation would be the presence of additional virulence factors in historical plague strains, which have been lost from current *Y. pestis* strains.

To answer this question, 2 ancient *Y. pestis* genomes were sequenced and compared with sequences of current *Y. pestis* isolates. The ancient genomes were from a pandemic European *Y. pestis pestis* biovar Antiqua isolate (genotype 1.ANT) that originated during the Black Death period (*3*), and from a pandemic *Y. pestis pestis* biovar Antiqua isolate (genotype 0.ANT), which was isolated from a patient who died during the plague of Justinian in Germany (*5*). Sequences of current *Y. pestis* genomes used for comparison were from the nonhuman pathogenic Chinese *Y. pestis microtus* strain (91001: 0.PE4, biovar Xilingolensis) and from the *Y. pestis pestis* strain (CO92: 1.ORI1, biovar Orientalis) (*3*,*5*).

Analyses showed that genomes of ancient *Y. pestis* strains did not contain additional virulence genes that might explain higher virulence. (*3**,**5*). Furthermore, because plague epidemics were caused by different biovars or genotypes in Asia, Europe, and Africa (*3*,*5*,*6*,*10*,*12*–*15*), we assume that various *Y. pestis* subtypes are similar in virulence. Thus, factors other than differences in virulence might better explain the decrease in the reported plague cases. It is more likely that the general perception and understanding of infectious agents; improved hygiene; trade management; knowledge of plague pathogenesis in humans; vector control; specific outbreak management, including selective isolation of infectious patients; and chemoprophylaxis and treatment with antimicrobial drugs, have accounted for the decrease in plague cases (*9*,*11*,*15*,*26*).

In conclusion, the 1.ORI3-k genotype of *Y. pestis* identified in the present study has virulence comparable with that of ancestral genotypes that caused other epidemics. The course and outcome of a human plague epidemic depend on this virulence, as well as on education, public awareness, life style, infrastructure, isolation of patients, and medical care. However, there is an unpredictable pathogenic potential in drug-resistant strains that has not been estimated and needs to be studied.
